# Association of central blood pressure with an exaggerated blood pressure response to exercise among elite athletes

**DOI:** 10.1007/s00421-023-05353-7

**Published:** 2023-11-21

**Authors:** Astrid Most, Lutz Kraushaar, Oliver Dörr, Stanislav Keranov, Sophie Hoelscher, Rebecca Weber, Ebru Akdogan, Vincent Groesser, Faeq Husain-Syed, Holger Nef, Christian W. Hamm, Pascal Bauer

**Affiliations:** 1 Department of Cardiology and Angiology, Justus Liebig University Giessen, 35390 Giessen, Germany; 2 Department of Internal Medicine, Member of the German Center for Lung Research, Universities of Giessen and Marburg Lung Center, Justus Liebig University Giessen, Giessen, Germany; 3 Adiphea GmbH, Werbach, Germany; 4 Department of Cardiology, Kerckhoff Heart and Thorax Center, Bad Nauheim, Germany; 5 German Center for Cardiovascular Research (DZHK), Rhein-Main Partner Site, Bad Nauheim, Germany

**Keywords:** SBP/MET slope, Elite athletes, Pulse wave analysis, Exaggerated blood pressure, Vascular function, Central blood pressure

## Abstract

**Purpose:**

The systolic blood pressure/workload (SBP/MET) slope was recently reported to be a reliable parameter to identify an exaggerated blood pressure response (eBPR) in the normal population and in athletes. However, it is unclear whether an eBPR correlates with central blood pressure (CBP) and vascular function in elite athletes.

**Methods:**

We examined 618 healthy male elite athletes (age 25.8 ± 5.1 years) of mixed sports with a standardized maximum exercise test. CBP and vascular function were measured non-invasively with a validated oscillometric device. The SBP/MET slope was calculated and the threshold for an eBPR was set at > 6.2 mmHg/MET. Two groups were defined according to ≤ 6.2 and > 6.2 mmHg/MET, and associations of CBP and vascular function with the SBP/MET slope were compared for each group.

**Results:**

Athletes with an eBPR (*n* = 180, 29%) displayed a significantly higher systolic CBP (102.9 ± 7.5 vs. 100 ± 7.7 mmHg, *p* = 0.001) but a lower absolute (295 ± 58 vs. 384 ± 68 W, *p* < 0.001) and relative workload (3.14 ± 0.54 vs. 4.27 ± 1.1 W/kg, *p* < 0.001) compared with athletes with a normal SBP/MET slope (*n* = 438, 71%). Systolic CBP was positively associated with the SBP/MET slope (*r* = 0.243, *p* < 0.001). In multiple logistic regression analyses, systolic CBP (odds ratio [OR] 1.099, 95% confidence interval [CI] 1.045–1.155, *p* < 0.001) and left atrial volume index (LAVI) (OR 1.282, CI 1.095–1.501, *p* = 0.002) were independent predictors of an eBPR.

**Conclusion:**

Systolic CBP and LAVI were independent predictors of an eBPR. An eBPR was further associated with a lower performance level, highlighting the influence of vascular function on the BPR and performance of male elite athletes.

## Introduction

The phenomenon of the “athlete's heart”, characterized by functional and structural changes in the right and left ventricle, has been described in elite athletes participating in mixed and endurance sports disciplines. (Fábián et al. 2022; Martinez et al. 2021; Albaeni et al. 2021). These physiological adaptations are considered beneficial to generate high performance levels (Pelliccia et al. 2021) and linked to lower arterial stiffness (Bauer et al. 2021b), lower blood pressure (BP) (Pelliccia et al. 2021; Caselli et al. 2017), and consequently, lower cardiovascular risk in athletic populations (Pelliccia et al. 2021).

Arterial hypertension, however, is the most common cardiovascular disease in athletes (Caselli et al. 2017; Pelliccia et al. 2021). The underlying mechanisms are not fully understood, but it is believed that the high amount of training and longer exposure to higher exercise-induced BP levels may contribute to this phenomenon (Schultz et al. 2013). Furthermore, extreme pulse pressure amplifications from central to brachial BP of up to 40 mmHg may be observed in athletes due to their enhanced vascular function (Berge et al. 2015; Herbert et al. 2014), leading to “spurious systolic hypertension” in some individuals, typically tall, young, and well-trained men (Hulsen et al. 2006; Eeftinck Schattenkerk et al. 2018). As a result, measurement of central blood pressure (CBP) and vascular function is recommended to identify individuals at increased cardiovascular risk (Hodson et al. 2016; Cheng et al. 2013). CBP and aortic pulse wave velocity are believed to be better indicators of cardiovascular risk than brachial BP (Hodson et al. 2016) and are independently correlated with organ damage (Wang et al. 2009; Roman et al. 2007). Additionally, previous studies have suggested that increased arterial stiffness precedes the development of hypertension, and CBP was found to be a significant predictor of new-onset hypertension (Sugiura et al. 2020). Vascular functional assessment can provide additional information for cardiovascular risk classification, and, recently, validated non-invasive oscillometric devices have been introduced to simplify clinical assessment of vascular functional impairment at rest (Trinkmann et al. 2021).

Since subclinical vascular impairment leads to an exaggerated BP response (eBPR) to exercise even in the absence of hypertension at rest (Miyai et al. 2021; Haarala et al. 2020; Thanassoulis et al. 2012), clinical exercise testing offers an additional diagnostic opportunity. It was shown that individuals with an eBPR to exercise are at increased risk of developing arterial hypertension and cardiovascular events (Percuku et al. 2019; Jae et al. 2019) in the future. This also applies to athletes (Caselli et al. 2019; Tahir et al. 2019; Keller et al. 2022), although data informing the definition of a normal or eBPR to exercise in athletes are scarce, despite clinical exercise testing being a key component of pre-participation screening (Pelliccia et al. 2021). The 2018 ESC guidelines (Williams et al. 2018) state that there is currently no consensus on a normal BPR during exercise due to inconsistent study results that focused on absolute peak systolic BP values.

Recently, a workload-indexed approach to classify the BPR to exercise in the general population was introduced by Hedman et al. (Hedman et al. 2019) and a threshold of 6.2 mmHg/MET was found to define an eBPR. A SBP/MET slope, expressed as the slope of systolic BP (SBP) in response to workload (metabolic equivalent of task, MET), of > 6.2 mmHg/MET was associated with a 27% higher risk of mortality over 20 years in males compared to those with a SBP/MET slope < 4.3 mmHg/MET (Hedman et al. 2019). The utility of the SBP/MET slope for pre-participation screening was shown by our group for male (Bauer et al. 2020, 2021b) and female elite athletes (Bauer et al. 2021a). An eBPR, defined as SBP/MET slope > 6.2 mmHg/MET, predicted left ventricular hypertrophy independent of age and sex in elite athletes referred for clinical evaluation in a pre-participation setting (Keller et al. 2022). CBP and vascular functional parameters have not yet been evaluated with the SBP/MET slope definition of an eBPR, and reference values for athletes are missing.

Therefore, we compared the functional and structural adaptations of male athletes (performing different mixed sports) with an eBPR with athletes with a normal BPR. We hypothesized that the former would display a higher CBP and worse vascular function compared with the latter and that certain vascular functional parameters may predict an eBPR. Hence, we investigated the significance of correlations between vascular functional parameters and the SBP/MET slope in male elite athletes. In addition, we assessed the influence of clinical and sports-related parameters as well as echocardiographic factors on the SBP/MET slope in our group of healthy male elite athletes.

## Materials and methods

### Study design

This was a single-center, cross-sectional registry study conducted at the university hospital of Giessen involving professional athletes during the routine pre-season medical monitoring program of the first German handball and basketball divisions and the second German handball, soccer, and ice hockey divisions. Data were collected in July and August of the years 2017–2022 after a 6-week competition-free interval. Only male athletes aged 18–39 were included in the study.

Subjective health status, medication, nutrition supplementation, amount of training, and history of training were assessed by questionnaire. Only individuals free of underlying cardiovascular diseases and medication were included. All tests were conducted at least 3 h post-prandially, and subjects refrained from exercise for at least 36 h prior to the test.

All participants provided their written informed consent. The local ethics committee of the University of Giessen approved the study protocol (AZ 15/17). The study was performed in accordance with the ethical standards laid down in the Declaration of Helsinki and its later amendments.

### Study population

The study enrolled a cohort of 618 male professional athletes of different ethnicities who participated in various sports disciplines such as handball, basketball, ice hockey, and soccer and hailed from different countries. All participants were non-smokers and did not take any medications or supplements regularly. A comprehensive physical examination, 12-lead electrocardiogram (ECG), resting BP measurements, and transthoracic echocardiography were performed on all individuals. The participants' age, height, weight, and body mass index (BMI) were recorded, and their body surface area (BSA) was calculated using the DuBois formula.

### Blood pressure measurement at rest

The study utilized a validated automatic device based on a standard sphygmomanometer technique (Boso clinicus, Bosch + Sohn GmbH & Co. KG, Germany) to measure resting brachial BP. The cuff used for measurements was adjusted to the individual’s arm circumference. A trained research associate performed measurements on both arms of the athlete while they were in a sitting position, following a resting period of 5 min, and repeated the measurements after 2 min. Athletes with a mean SBP ≥ 140 mmHg or diastolic BP (DBP) ≥ 90 mmHg were excluded from the study.

### Echocardiography

All athletes were examined by standard transthoracic echocardiography administered by an experienced cardiologist according to the current recommendations (Lang et al. 2015; Baggish et al. 2020) using a Philips cx50 echocardiography system (Philips, Eindhoven, The Netherlands) with the participant in a left lateral supine position. Standard measurements of cardiac dimensions, contractility, and diastolic function were obtained. Each parameter was assessed in three–five consecutive cardiac cycles, and mean values were used for data recording and analysis.

Left ventricular (LV) wall thicknesses and diameters were evaluated in the parasternal long-axis view at the level of mitral valve coaptation. Further, volumes and ejection fraction (EF) were determined using Simpson’s biplane method. LV stroke volume was calculated as the product of LV outflow tract area and outflow tract time–velocity integral, and right ventricular (RV) stroke volume was calculated similarly as the product of RV outflow tract area and outflow tract time–velocity integral.

LV mass was calculated using the Devereux formula and indexed to body surface area to obtain the LV mass index (LVMI). LV hypertrophy was defined as an LVMI > 115 g/m^2^. Left atrial volume index (LAVI) was obtained by the area–length method.

Peak tricuspid regurgitant velocity (TRV) was measured from the spectral profile of the tricuspid regurgitation jet in the RV inflow projection of the parasternal short-axis view or the apical four-chamber view. Pulmonary artery systolic pressure (SPAP) was then calculated based on the simplified Bernoulli equation applied to TRV by adding a value of right atrial pressure as measured by inferior vena cava respiratory index to the systolic trans-tricuspid gradient. SPAP was assumed to equate the RV systolic pressure in the absence of pulmonary stenosis and/or RV outflow tract obstruction.

Tricuspid annular plane systolic excursion (TAPSE) was measured from the four-chamber views by placing an M-mode cursor through the tricuspid annulus and measuring the excursion distance in mm between end-diastole and end-systole.

### Non-invasive assessment of vascular function

The vascassist2^®^ device, developed by isymed GmbH (Butzbach, Germany), was used to collect pulse pressure waveforms through oscillometry in a non-invasive manner. This device uses a validated model of the arterial tree consisting of 721 electronic circuits that mimic an individual's pulse pressure waves by adjusting the circuits' capacitance, resistance, inductance, and voltage (Schumacher et al. 2018; Trinkmann et al. 2021). The system uses evolutionary algorithms to optimize the fidelity of the pulse pressure wave replication, ensuring that fidelity replications of 99.6% or higher are included in the analysis.

All participants underwent non-invasive vascular evaluation after resting for 15 min in a supine position. The evaluation was performed using four conventional cuffs adapted to the upper arm and forearm circumferences of each participant. Radial and brachial pulse pressure waves were obtained on both arms with step-by-step deflation of the cuffs. The measurements were conducted in a room with a comfortable and stable temperature of 22 °C and no external stress influences. Participants were instructed to remain still during the pulse pressure wave acquisition, and stable and valid results were ensured through the performance of two brachial and three radial measurements, with a 30-s break between each measurement phase. The total duration of the examination was 15 min.

The acquired pulse pressure waves were then analyzed with a validated electronic model of the arterial tree to assess vascular functional parameters. Brachial and radial SBP and DBP, CBP, aortic pulse wave velocity (PWV), augmentation index (Aix), augmentation index at a heart rate of 75 bpm (Aix@75), resistance index (R), total vascular resistance, and ejection duration were calculated. CBP was determined using a validated transfer function based on the peripheral arterial waveform, and calculation of Aix@75 was also based on the pulse waveform.

### Exercise testing

Athletes underwent a standardized progressive maximal cycling ergometer test while their brachial BP and ECG were automatically measured and recorded (Schiller AG^®^, Switzerland). The test began with a warm-up period of 2 min at 50 W that was followed by a load level of 100 W that was increased by 50 W every 2 min until exhaustion, which was defined as the participant’s inability to maintain the load for 2 min. The test ended with a decrease in load to 25 W for 3 min of active recovery, followed by a 2-min cool-down period at rest. The test concluded with a final ECG recording and a brachial BP measurement. BP was measured at every stage during test and recovery periods, including at the maximum workload, immediately after the maximum workload, immediately after the end of the test, and after 5 min of recovery. Heart rate was measured with continuous ECG recording throughout the test and recovery periods. The absolute maximum workload of the athletes as well as the workload adjusted to individual body weight were assessed. Other measurements included maximum heart rate and heart rate at rest and 5 min after the exercise test. Increases in SBP and DBP were calculated from peak and baseline (resting) values. Pulse pressure was calculated as SBP–DBP at rest and at maximum exercise. In addition, mean BP was determined as: DBP + (SBP − DBP)/3.

MET was estimated using standard equations of the American Council of Sports Medicine (ACSM) for cycling ergometers (Thompson et al. 2013). The ΔSBP was calculated as (maximum SBP–SBP at rest) and indexed by the increase in MET from rest (ΔMET calculated as peak MET − 1) to obtain the SBP/MET slope. Based on their SBP/MET slope, athletes were allocated to either of two groups: the first group was defined as normal BPR to exercise with SBP/MET slope ≤ 6.2 mmHg/MET, and the second group was classified as eBPR with SBP/MET slope > 6.2 mmHg/MET.

### Statistical analysis

Descriptive analyses were carried out on all study variables for the total sample and separated by SBP/MET slope (≤ 6.2 mmHg/MET and > 6.2 mmHg/MET). All data are presented as mean ± standard deviation (SD). The Shapiro–Wilk test was used to determine normal distribution. If the data were determined to have a skewed distribution, all analyses were performed on normalized data. Between-group comparisons were made using independent sample *t* tests. The Pearson partial correlation test was used to assess the relationship between central and brachial BP values, arterial stiffness, vascular function, SBP/MET slope, and clinically relevant variables such as age, BMI, BSA, training history, amount of training per week, LV stroke volume, LAVI, heart rate, *E*/*A*, and *E*/*E*′.

Bivariate and multiple logistic regression models were used to test the weight of hemodynamic parameters and age on an eBPR, defined as SBP/MET slope > 6.2 mmHg/MET. Results of the logistic regressions are presented as odds ratio (OR) and 95% confidence interval (CI).

The two-tailed significance level was set at *p* < 0.05 for all measurements. All statistical analyses were performed using the IBM SPSS Statistics for Macintosh, Version 27.0 (IBM Corp., Armonk, NY, USA).

## Results

### Cohort characteristics

All 618 male elite athletes included in the study were participants in mixed team sports disciplines that are characterized by a high-intensity level (handball, ice hockey, soccer and basketball) (Pelliccia et al. 2021). The mean age of the participants was age 25.8 ± 5.1 years; the mean height was 188.7 ± 8.5 cm and weight 95.5 ± 12.2 kg, resulting in a BMI of 25.6 ± 1.9 kg/m^2^ (Table 1). The probands were experienced athletes who had participated in professional training for 8.95 ± 5.2 years with a current mean training time of 18.5 ± 3.7 h per week.

**Table 1:** Clinical characteristics of the cohort athletes according to defined SBP/MET slope cut-off

	Male elite athletes		*p* value
	SBP/MET slope ≤ 6.2 mmHg/MET	SBP/MET slope > 6.2 mmHg/MET	
Number (%)	438 (71)	180 (29)	
Age (years)	26.2 ± 5.2	25.6 ± 5.1	0.123
Height (cm)	189.3 ± 7.6	190 ± 7.6	0.166
Body weight (kg)	92 ± 10.7	94.4 ± 11.7	**0.019**
Body mass index (kg/m^2^)	25.6 ± 1.8	26 ± 2.1	**0.022**
Body surface area (m^2^)	2.2 ± 0.17	2.2 ± 0.17	**0.037**
Training history (years)	9.3 ± 5.3	8.7 ± 5.2	0.260
Training per week (hours)	18.8 ± 3.2	19.3 ± 2.6	0.062
Systolic blood pressure (mmHg)	124.7 ± 11.2	124.6 ± 9.8	0.906
Diastolic blood pressure (mmHg)	63.5 ± 9.6	64.9 ± 9.5	0.164
Mean arterial blood pressure (mmHg)	80 ± 9.6	80.7 ± 9.5	0.553
Resting heart rate (/min)	58.7 ± 10.7	56.4 ± 9.3	**0.008**

Values are expressed as mean ± SD

Bold values denote statistical significance at the *p* < 0.05 level

Athletes with an SBP/MET slope ≤ 6.2 mmHg/MET displayed a lower body weight (92 ± 10.7 vs. 94.4 ± 11.7 kg, *p* = 0.019), BMI (25.6 ± 1.8 vs. 26 ± 2.1 kg/m^2^, *p* = 0.022), and BSA (2.19 ± 0.17 vs. 2.23 ± 0.17 m^2^, *p* = 0.037) compared to those with SBP/MET slope > 6.2 mmHg/MET. The clinical characteristics, anthropometric data, and specific training data are displayed in detail in Table 1.

The mean systolic and diastolic BP values of the entire study cohort were 124.7 ± 10.8 mmHg and 63.8 ± 8.9 mmHg, respectively. There were no significant differences in SBP between athletes with an SBP/MET slope ≤ 6.2 mmHg/MET (124.7 ± 11.2 mmHg) and those with an SBP/MET slope > 6.2 mmHg/MET (124.6 ± 9.8 mmHg). None of the included athletes had an SBP ≥ 140 mmHg or DBP ≥ 90 mmHg. Athletes with an SBP/MET slope > 6.2 mmHg/MET displayed a significantly lower heart rate at rest compared with their peers with an SBP/MET slope ≤ 6.2 mmHg/MET (56.4 ± 9.3 vs. 58.7 ± 10.7 bpm, *p* = 0.008).

### Vascular functional and central blood pressure measurements

There were no significant differences in aortic pulse wave velocity, augmentation pressure, ejection duration, augmentation index@75 bpm (Aix@75), total peripheral resistance, mean aortic BP and central DBP between the two groups. In contrast, there were significant differences in central SBP, with higher values in athletes assigned to the > 6.2 mmHg/MET group (102.9 ± 7.5 vs. 100.2 ± 7.7 mmHg, *p* = 0.001).

### Echocardiographic characteristics

Echocardiographic characteristics are summarized in Table 2. The two groups differed significantly in the LVEF (*p* = 0.030), left atrial diameter (*p* = 0.013), left atrial volume index (*p* < 0.001), and LV end-systolic diameter (*p* = 0.034), with athletes assigned to the > 6.2 mmHg/MET group displaying higher values for these parameters.

**Table 2:** Vascular function, echocardiographic, and exercise testing results of the cohort athletes according to defined SBP/MET slope cut-off

	Male elite athletes		*p* value
	SBP/MET slope ≤ 6.2 mmHg/MET	SBP/MET slope > 6.2 mmHg/MET	
Central blood pressure and vascular function			
Systolic central BP (mmHg)	100.2 ± 7.7	102.9 ± 7.5	**0.001**
Diastolic central BP (mmHg)	62.5 ± 9.7	64.6 ± 10.5	0.058
Mean aortic BP (mmHg)	77.7 ± 8.8	79.5 ± 10.1	0.148
Aortic pulse wave velocity (m/s)	6.39 ± 1.47	6.34 ± 1.66	0.398
Augmentation index @75 bpm (%)	− 20.3 ± 10.3	− 21.4 ± 10.5	0.322
Augmentation pressure (mmHg)	− 5.27 ± 3.8	− 5.34 ± 4.1	0.869
Ejection duration (ms)	300.7 ± 31.4	301.5 ± 24.8	0.793
Total peripheral resistance (dyn*s/cm^5^)	1386 ± 382	1424 ± 424	0.553
Pulse pressure amplification (mmHg)	25.74 ± 5.89	25.8 ± 6.0	0.867
Echocardiographic parameters			
LV ejection fraction (%)	66.2 ± 4.6	67.1 ± 4.5	**0.030**
LV stroke volume (mL)	93.2 ± 18.5	91.5 ± 15.8	0.337
LV end-diastolic diameter (mm)	53.9 ± 3.6	53.5 ± 3.8	0.193
LV end-systolic diameter (mm)	33.6 ± 3.4	32.9 ± 3.6	**0.034**
Left atrial diameter (mm)	35.4 ± 3.1	37.3 ± 3.3	**0.013**
Left atrial volume index (ml/m^2^)	24.6 ± 4.1	27.8 ± 3.7	**< 0.001**
Septal wall thickness (mm)	10.19 ± 1	10.29 ± 1.2	0.360
Inferior wall thickness (mm)	9.8 ± 0.9	9.9 ± 1.2	0.134
LV mass index (g/m^2^)	87.8 ± 26	90.1 ± 24.8	0.329
Relative wall thickness (%)	33 ± 8	34 ± 6	0.452
*E*/*A* ratio	1.84 ± 0.42	1.83 ± 0.43	0.908
*E*/*E*′ lateral	5.27 ± 1.3	5.43 ± 1.32	0.223
*E*/*E*′ medial	6.82 ± 1.17	6.7 ± 1.41	0.301
*E*/*E*′ average	6.2 ± 1	6.25 ± 1.31	0.352
RV diameter 1 (mm)	38.5 ± 5.1	39.1 ± 6.2	0.094
RVOT PLAX (mm)	29.2 ± 1.9	30.5 ± 2.1	0.138
RV stroke volume (mL)	91.9 ± 14.7	90.6 ± 16.2	0.672
Fractional area change (%)	50.6 ± 6.1	51.2 ± 6.4	0.074
RV *s*′ (cm/s)	15.3 ± 2.3	15.8 ± 1.9	0.308
TAPSE (mm)	29 ± 4.3	29.8 ± 4.6	0.074
SPAP (mmHg)	20 ± 4.7	19.3 ± 4.4	0.078
TASPSE/SPAP (mm/mmHg)	1.26 ± 0.32	1.32 ± 0.34	0.051
Exercise testing			
Systolic blood pressure at rest (mmHg)	126.3 ± 8.4	129 ± 7.6	**0.002**
Diastolic blood pressure at rest (mmHg)	76.8 ± 8.2	75.5 ± 8.1	0.089
Heart rate at rest (bpm)	61.3 ± 11.8	59 ± 9	0.085
Absolute workload (W)	384.3 ± 68.6	295.3 ± 58.5	**< 0.001**
Relative workload (W/kg)	4.27 ± 1.1	3.14 ± 0.54	**< 0.001**
Peak energy expenditure (MET)	16.6 ± 3.9	12.6 ± 2	**< 0.001**
Max. systolic BP (mmHg)	188.8 ± 17.5	213.3 ± 14.6	**< 0.001**
Max. diastolic BP (mmHg)	83.4 ± 9.4	85.2 ± 8.6	**0.024**
Max. heart rate (bpm)	178.6 ± 10	179.2 ± 11.5	0.540
Max. heart rate (% of calculated max. heart rate)	94.2 ± 5.1	94.2 ± 5.9	0.885
Rating of perceived exertion (Borg scale)	18.7 ± 0.5	18.5 ± 0.7	0.512
SBP/MET slope (mmHg/MET)	4.26 ± 1.16	7.72 ± 1.29	**< 0.001**

Values are expressed as mean ± SD

Bold values denote statistical significance at the *p* < 0.05 level

*BP* blood pressure, *bpm* beats per minute, *BMI* body mass index, *LV* left ventricle, *PLAX* parasternal long-axis view, *RV* right ventricle, *RVOT* right ventricular outflow tract, *SPAP* systolic pulmonary artery pressure, *TAPSE* tricuspid annular plane systolic excursion

### Exercise testing results

As expected, athletes with an eBPR displayed a higher SBP/MET slope compared with those with a normal BPR (7.72 ± 1.29 vs. 4.26 ± 1.16 mmHg/MET, *p* < 0.001). Further, these athletes had a higher systolic BP at the beginning of the test (*p* = 0.002) and a higher maximum systolic (*p* < 0.001) and diastolic BP (*p* < 0.001) compared with those with SBP/MET slope ≤ 6.2 mmHg/MET. In contrast, athletes with an eBPR achieved a lower absolute (*p* < 0.001) and relative (*p* < 0.001) workload and had, correspondingly, a lower peak energy expenditure (*p* < 0.001) compared with those with a normal BPR.

### Prevalence of eBPR

Application of a cut-off of 6.2 mmHg/MET in the SBP/MET slope to differentiate a normal from an exaggerated BPR resulted the classification of 180 athletes (29%) as eBPR and 438 (71%) as normal BPR. Athletes with an eBPR displayed a maximum SBP of 213.3 ± 14.6 mmHg. In the eBPR group, the lowest maximum SBP was 190 mmHg and the highest was 264 mmHg, with range of SBP/MET slope from 6.21 to 9.8 mmHg/MET. In the group with a normal BPR, the lowest measured maximum SBP was 168 mmHg and the highest was 223 mmHg, respectively. The corresponding range in SBP/MET slope was 3.2–6.2 mmHg/MET.

### Association of an exaggerated blood pressure response to exercise with clinical, echocardiographic, and vascular parameters

An eBPR was positively correlated with mean aortic BP (*r* = 0.226, *p* = 0.038). No other significant correlations of the investigated parameters with an eBPR were found. Pearson’s partial correlation test results for the clinical and echocardiographic parameters are presented in Table 3.

**Table 3:** Pearson correlation coefficients for the association between an SBP/MET slope > 6.2 mmHg/MET and various vascular and echocardiographic parameters

	SBP/MET slope > 6.2 mmHg/MET	
	*r*	*p*
Age (years)	0.037	0.623
Height (cm)	0.135	0.073
BSA (m^2^)	0.143	0.081
Training history (years)	0.008	0.915
Training per week (h)	− 0.024	0.758
Heart rate (/min)	− 0.109	0.147
Central systolic blood pressure (mmHg)	0.151	0.097
Central diastolic blood pressure (mmHg)	0.162	0.075
Mean aortic blood pressure (mmHg)	0.226	**0.038**
Aortic augmentation pressure (mmHg)	0.013	0.886
Aortic pulse wave velocity (m/s)	− 0.010	0.916
Augmentation index @75/min (%)	− 0.035	0.702
Peripheral resistance (dyn*s/cm^5^)	− 0.110	0.120
Brachial systolic blood pressure (mmHg)	0.040	0.592
Brachial diastolic blood pressure (mmHg)	− 0.045	0.550
Maximum heart rate (/min)	0.137	0.069
LV ejection fraction (%)	− 0.041	0.597
LV end-diastolic diameter (mm)	0.092	0.231
LV end-systolic diameter (mm)	0.067	0.386
Left atrial diameter (mm)	0.028	0.716
Left atrial volume index (ml/m^2^)	0.033	0.851
Septal wall thickness (mm)	0.020	0.801
Inferior wall thickness (mm)	− 0.039	0.617
LV mass index (g/m^2^)	0.032	0.673
RV diameter 1 (mm)	0.076	0.329
Fractional area change (%)	− 0.033	0.851
TAPSE (mm)	0.119	0.126
E/A	− 0.058	0.488
*E*/*E*′ lat	− 0.005	0.948
*E*/*E*′ med	0.004	0.961
*E*/*E*′ average	0.002	0.972

Bold values denote statistical significance at the *p* < 0.05 level

*BMI* body mass index, *BSA* body surface area, *LV* left ventricle, *SPAP* systolic pulmonary artery pressure, *TAPSE* tricuspid annular plane systolic excursion

### Clinical, echocardiographic, and vascular functional predictors of an eBPR

Bivariate and multivariate logistic regression analyses were performed to identify the association between eBPR with the central and peripheral BP values at rest (Tables 4 and 5). In bivariate analyses (Table 4), LVEF (OR 1.048, CI 1.008–1.090, *p* = 0.020), LV end-systolic diameter (LVES; OR 0.941, CI 0.894–0.992, *p* = 0.023), aortic root size (OR 1.089, CI 1.026–1.156, *p* = 0.005), TAPSE (OR 1.046, CI 1.005–1.090, *p* = 0.029), LAVI (OR 1.243, CI 1.101–1.404, *p* = 0.002) and systolic CBP (OR 1.032, CI 1.008–1.057, *p* = 0.009) were significantly associated with an eBPR.

**Table 4:** Bivariate logistic regression analyses with SBP/MET slope > 6.2 mmHg/MET as dependent variable

	Regression coefficient	SE	Wald	OR (95% CI)	*p*
LVEF	0.047	0.02	5.446	1.048 (1.008–1.090)	**0.020**
LVEDD	− 0.030	0.024	1.602	0.970 (0.925–1.017)	0.206
LVES	− 0.060	0.27	5.157	0.941 (0.894–0.992)	**0.023**
Ao	0.085	0.30	7.836	1.089 (1.026–1.156)	**0.005**
TAPSE	0.045	0.021	4.742	1.046 (1.005–1.090)	**0.029**
RV	− 0.024	0.015	2.447	0.976 (0.947–1.006)	0.118
FAC	0.015	0.034	0.189	1.015 (0.949–1.085)	0.664
*s*′	0.094	0.097	0.951	1.099 (0.909–1.329)	0.330
LAVI	0.218	0.062	12.256	1.243 (1.101–1.404)	**0.002**
*E*/*A*	− 0.079	0.231	0.116	0.924 (0.587–1.454)	0.733
*E*/*E*′ lat	0.069	0.077	0.783	1.071 (0.920–1.246)	0.376
*E*/*E*′ med	− 0.107	0.074	2.073	0.899 (0.808–0.884)	0.150
Septal wall thickness	0.103	0.083	1.554	1.109 (0.943–1.304)	0.213
Inferior wall thickness	0.086	0.089	0.944	1.090 (0.916–1.298)	0.331
LVMI	− 0.004	0.003	1.208	0.996 (0.989–1.003)	0.272
PWV	− 0.073	0.092	0.635	0.929 (0.776–1.113)	0.426
TPR	− 0.015	0.017	0.793	0.985 (0.953–1.018)	0.373
sCBP	0.032	0.012	6.897	1.032 (1.008–1.057)	**0.009**
dCBP	0.016	0.010	2.447	1.016 (0.996–1.036)	0.118
Mean CBP	0.014	0.012	1.300	1.014 (0.990–1.038)	0.254
Aix@75	− 1.259	1.004	1.573	0.284 (0.040–2.030)	0.210
sBBP	0.004	0.008	0.199	1.004 (0.988–1.020)	0.656
dBBP	− 0.016	0.110	2.131	0.984 (0.963–1.005)	0.144
Mean BBP	− 0.015	0.012	1.591	1.011 (0.986–1.037)	0.399

Bold values denote statistical significance at the *p* < 0.05 level

*Aix@75* augmentation index corrected to 75 beats per minute, *dBBP* diastolic brachial blood pressure, *dCBP* diastolic central blood pressure, *CBP* central blood pressure, *BPP* brachial blood pressure, *FAC* fractional area shortening, *LAVI* left atrial volume index, *LV* left ventricle, *LVEDD* left ventricular end-diastolic diameter, *LVES* left ventricular end-systolic diameter, *PWV* pulse wave velocity, *RV* right ventricle, *sBBP* systolic brachial blood pressure, *sCBP* systolic central blood pressure, *SPAP* systolic pulmonary artery pressure, *TAPSE* tricuspid annular plane systolic excursion, *TPR* total peripheral resistance

**Table 5:** Multiple logistic regression analyses with SBP/MET slope > 6.2 mmHg/MET as dependent variable

	Regression coefficient	SE	Wald	OR (CI)	*p*
**Model 1**					
PWV	− 0.097	0.102	0.905	0.908 (0.745–1.108)	0.341
TPR	− 0.016	0.020	0.702	0.984 (0.947–1.022)	0.402
sCBP	0.094	0.025	13.693	1.099 (1.045–1.155)	**< 0.001**
dCBP	− 0.020	0.019	1.108	0.980 (0.945–1.077)	0.293
Aix@75	− 1.156	1.215	0.905	0.341 (0.029–3.404)	0.905
SBBP	− 0.100	0.012	0.728	0.990 (0.967–1.013)	0.393
dBBP	− 0.021	0.015	2.763	0.969 (0.942–1.010)	0.065
Constant	− 4.868	1.910	6.498		0.110
**Model 2**					
LVEF	− 0.065	0.087	0.563	0.937 (0.790–1.111)	0.453
LVEDD	0.031	0.164	0.035	1.031 (0.748–1.422)	0.851
LVES	− 0.070	0.147	0.230	0.932 (0.699–1.243)	0.632
Ao	0.105	0.122	0.739	1.110 (0.875–1.410)	0.390
TAPSE	0.034	0.063	0.288	1.034 (0.915–1.169)	0.591
RV	0.152	0.129	1.399	1.164 (0.905–1.499)	0.237
FAC	0.019	0.050	0.139	1.019 (0.923–1.125)	0.709
*s*′	0.040	0.145	0.076	1.041 (0.783–1.384)	0.782
LAVI	0.248	0.081	9.516	1.282 (1.095–1.501)	**0.002**
*E*/*A*	0.455	0.771	0.348	1.576 (0.348–7.144)	0.555
*E*/*E*′ lat	0.425	0.328	1.680	1.530 (0.804–2.911)	0.195
*E*/*E*′ med	− 0.099	0.313	0.101	0.906 (0.491–1.671)	0.751
Septal wall thickness	0.876	0.668	1.719	2.401 (0.648–8.896)	0.190
Inferior wall thickness	0.121	0.582	0.043	1.128 (0.360–3.534)	0.836
LVMI	− 0.096	0.049	3.811	0.908 (0.825–1.000)	0.051
Constant	− 17.869	10.869	2.754		0.097

Degrees of freedom were 1 for all Wald statistics

In Model 1, PWV, TPR, systolic CBP, diastolic CBP, Aix@75, brachial SBP and brachial DBP were used as independent parameters. In Model 2, LVEF, LVEDD, LVES, LVMI, aortic root size, TAPSE, RV diameter 1, *s*′, FAC, LAVI, E/A, *E*/*E*′ med., and *E*/*E*′ lat. served as independent parameters

Bold values denote statistical significance at the *p* < 0.05 level

*Aix@75* augmentation index corrected to 75 beats per minute, *dBBP* diastolic brachial blood pressure, *dCBP* diastolic central blood pressure, *CBP* central blood pressure, *BPP* brachial blood pressure, *FAC* fractional area shortening, *LAVI* left atrial volume index, *LV* left ventricle, *LVEDD* left ventricular end-diastolic diameter, *LVES* left ventricular end-systolic diameter, *PWV* pulse wave velocity, *RV* right ventricle, *sBBP* systolic brachial blood pressure, *sCBP* systolic central blood pressure, *TAPSE* tricuspid annular plane systolic excursion, *TPR* total peripheral resistance

Multiple logistic regression models were used to determine the effect of central hemodynamic and vascular functional parameters (Model 1) and various echocardiographic parameters (Model 2) to predict the likelihood of an exaggerated blood pressure response, measured as SBP/MET slope > 6.2 mmHg/MET.

Linearity was tested using the Box–Tidwell procedure. Bonferroni correction was applied to all seven terms in model 1 and to all fifteen terms in model 2. All variables were found to follow a linear relationship.

Correlations between predictor variables were low (*r* < 0.70) in both models, indicating that multicollinearity was not a confounding factor in the analysis.

Goodness-of-fit was assessed using the Hosmer–Lemeshow Test, indicating a good model fit for both model 1 (*χ*^2^(8) = 10.21, *p* = 0.251) and for model 2 (*χ*^2^(8) = 7.16, *p* = 0.519).

Model 1 examined the effects of the vascular functional and central hemodynamic parameters PWV, total peripheral resistance, systolic CBP, diastolic CBP, Aix@75, brachial SBP and brachial DBP as independent parameters on an eBPR, and was statistically significant, *χ*^2^(7) = 27.32, *p* < 0.001, resulting in a low amount of explained variance, as shown by Nagelkerke’s *R*^2^ = 0.091. Of the seven variables entered into the logistic regression model, only the systolic CBP (OR 1.099, CI 1.045–1.155, *p* < 0.001) was a significant predictor of an eBPR, while the other variables showed no significant effect.

Model 2 examined the effects of the echocardiographic parameters LVEF, LVEDD, LVES, LVMI, aortic root size, TAPSE, RV diameter 1, *s*′, FAC, LAVI, E/A, *E*/*E*′ med. and *E*/*E*′ lat. as independent parameters on an eBPR and was also statistically significant, *χ*^2^(15) = 34.269, *p* = 0.003, resulting in a moderate amount of explained variance, as shown by Nagelkerke’s *R*^2^ = 0.402. Of the fifteen variables entered into the regression model, only LAVI (OR 1.282, CI 1.095–1.501, *p* = 0.002) contributed significantly in predicting an eBPR, while the other variables showed no significant effect. The detailed results of the analyses are presented in Table 5.

## Discussion

Our study is the first to comprehensively evaluate CBP and cardiovascular function in healthy male elite athletes participating in various sports and to examine its correlation with the workload-indexed BP response to a standardized maximum exercise test, measured as the SBP/MET slope. Our main findings are:

1. the systolic CBP at rest was associated with the SBP/MET slope in male elite athletes;
2. athletes with an eBPR displayed a significantly higher systolic CBP, but not diastolic CBP or brachial BP, compared with athletes with a normal BPR;
3. an eBPR was associated with a lower physical performance;
4. LAVI and systolic CBP were significant predictors of an eBPR.

The clinical significance of BPR in athletes is not yet clear, although it has been suggested that athletes with an exaggerated BPR may be at higher risk of developing arterial hypertension (Caselli et al. 2019) as well as myocardial fibrosis (Tahir et al. 2018), raising concerns about potential arrhythmic consequences, including sudden cardiac death (Zorzi et al. 2016). Defining the upper limits of normal BPR and considering specific workload, instead of using absolute thresholds, may help to distinguish normal from exaggerated BPR. Recently, Hedman et al. (Hedman et al. 2019) found an increase in systolic BP per increase in metabolic equivalent of task (SBP/MET slope) > 6.2 mmHg/MET to be associated with a 27% higher risk of mortality over 20 years in males (mean age 59 years) with a high fitness level (achieving ≥ 8.2 MET in an exercise test) compared with those with a SBP/MET slope of < 4.3 mmHg/MET. The low-risk group in the study by Hedman et al. (2019) included non-smoking subjects surviving at least 10 years following the exercise test, without a history of diabetes mellitus, hypertension or cardiovascular disease. The authors concluded that the calculated SBP/MET slope, as a workload- indexed measure of systolic BPR to exercise, may add prognostic precision in subjects with higher fitness levels (Hedman et al. 2019).

Our previous studies have shown that the SBP/MET slope could be used in pre-participation screening for elite male (Bauer et al. 2020) and female (Bauer et al. 2021a) athletes to identify athletes at risk of an eBPR.

Keller et al. (2022) recently investigated the SBP/MET slope in a large group of elite athletes of both sexes and compared the threshold of > 6.2 mmHg/MET used to define an eBPR with earlier proposed thresholds that used absolute maximum systolic BP values. Interestingly, the authors found that an SBP/MET slope > 6.2 mmHg/MET was significantly associated with concentric remodeling and concentric hypertrophy of the left ventricle, higher LV mass and larger left atrial area. These findings highlight the potential of the SBP/MET slope to identify an eBPR in athletes and point toward the possibility for an early detection of athletes at risk.

In contrast, our study did not find an association between an eBPR and LV hypertrophy in our homogeneous male cohort of elite athletes, which may be due to differences in study cohorts and the definition of LV hypertrophy (Keller et al. 2022). Consistent with the study by Keller et al. (2022), we found that LAVI was associated with an eBPR and served as an independent predictor in our study cohort. Our findings suggest that athletes with an eBPR had a measurable diastolic dysfunction, as evidenced by significantly higher LAVI than in those without an eBPR. However, there were no significant differences in other LV diastolic functional parameters between the two groups. Furthermore, PWV, as an acknowledged marker of arterial stiffness (Ben-Shlomo et al. 2014; Thijssen et al. 2016; Vlachopoulos et al. 2019), was not different between the groups. Another study that investigated arterial stiffness and diastolic function also reported no association between brachial–ankle pulse wave velocity (baPWV) and LAVI (Kim et al. 2022).

Central hemodynamic parameters and vascular function are known to play a crucial role in BP regulation (Currie et al. 2019; Bauer et al. 2019a; Yu et al. 2018; Sun et al. 2018; Safar 2018; Green and Smith 2018; Stephen Hedley and Phelan 2017; Ashor et al. 2014) and physical performance (Bauer et al. 2019b; Perissiou et al. 2018; Denham et al. 2016), not only in the general population (Pierce et al. 2018), but also in athletes of different sports (Sotiriou et al. 2019; Sardeli et al. 2018; Franzen et al. 2016). Although CBP is considered to be a more significant predictor of cardiovascular outcomes than brachial BP (Williams et al. 2006; Roman et al. 2007; Sugiura et al. 2020; Sun et al. 2018; Hodson et al. 2016; Fan et al. 2016), there are currently no normative values for CBP in athletes (Herbert et al. 2014). CBP is determined by the complex interaction between aortic compliance, elasticity, and the ability of resistance arteries to channel blood flow according to tissue needs (Stephen Hedley and Phelan 2017). Decreased distensibility of the central elastic arteries compromises their ability to buffer the ejected blood volume from the left ventricle, leading to an increase in CBP and compromising coronary flow (Thijssen et al. 2016). Elevated peripheral resistance resulting from increases in vessel constriction may amplify CBP elevations (Ashor et al. 2014). These factors may also contribute to a lower physical performance level, especially in highly trained athletes.

Functional vascular impairment might lead to an eBPR even in the absence of hypertension at rest (Miyai et al. 2021; Thanassoulis et al. 2012), and the hemodynamic effects of this impairment might be amplified during exercise. Thus, vascular functional assessment might provide additional information for cardiovascular risk classification. Similarly, another study by Haarala et al. (2020) found that arterial stiffness, measured as PWV at rest, was able to predict an eBPR in young and healthy individuals (*n* = 209, mean age 38 years, 49% males, 51% females). However, in contrast to this latter study but in line with our previous findings in elite athletes (Bauer et al. 2021b), our current findings did not show any significant association between PWV and eBPR in elite athletes. This difference could be attributed to our study cohort, which consisted of professional athletes who had a lower PWV and were significantly younger. In addition, Haarala et al. (2020) did not present their data separately for males and females, which limits comparison to our own results. Hence, our measured PWV values were consistent with previous studies and meta-analyses that analyzed elite athletes of different sports (Vlachopoulos et al. 2010; Ashor et al. 2014; Sardeli et al. 2018; Hashimoto and Okamoto 2020).

CBP has been shown to predict the development of hypertension in the general population (Sugiura et al. 2020). In our study, CBP was associated with the SBP/MET slope, and systolic CBP was able to predict an eBPR in our cohort of male healthy elite athletes across different sports. This is a clinically relevant finding, as an eBPR is considered to be a precursor of future arterial hypertension (Caselli et al. 2019). Given the high training levels of our investigated athletes, the repeated exposure to an eBPR over time and during an athletic career may accumulate and lead to hypertension-related organ damage. Therefore, particularly in young and trained males, the measurement of CBP is recommended to exclude isolated systolic hypertension (Eeftinck Schattenkerk et al. 2018; Harbin et al. 2018; Wilkinson et al. 2001). Notably, our study cohort displayed normal brachial BP values at rest without differences between the two groups. However, we observed significantly higher systolic CBP in athletes with an eBPR than in those with a normal BPR. This highlights the influence of vascular function and vascular adaptations to regular exercise in athletes.

In our cohort, CBP was lower than that of the general population, which is consistent with our previous studies dealing with elite athletes (Bauer et al. 2019a, 2021a, b) and with other studies examining elite athletes across different sports (Tomschi et al. 2021; Sotiriou et al. 2019; Sardeli et al. 2018; Franzen et al. 2016). Despite the fact that the CBP we determined was lower than the reported reference values for the general population, we detected a significantly lower physical performance level in the group with an eBPR, pointing toward a clinically relevant issue not only for future risk prediction, but also for the current performance level. The influence of BP on performance levels, even with BP below the currently recommended threshold for defining hypertension in Europe (Williams et al. 2018), has already been demonstrated in elite athletes (Mazic et al. 2015). This highlights the influence of altered central hemodynamic and vascular function on exercise capacities. Therefore, it is reasonable to speculate that vascular functional parameters differ during exercise conditions and lead to the detected differences in SBP/MET slope. Athletes with a normal BPR might display a higher arterial vasodilator reserve compared with those with an eBPR. Unfortunately, vascular functional parameters and CBP could not be measured during exercise to substantiate this hypothesis.

Taken together, these results highlight the problems inherent with the use of non-invasive devices that evaluate vascular function via oscillometry (Miyata 2018). These validated methods deliver reliable results at rest, but owing to the measurement technique, not during an exhaustive exercise test (Miyata 2018). Aside from their direct clinical implications, the observations that (a) BP response to exercise associates with future disease and event risk and that (b) the eBPR associates with measurable functional biomarkers at rest are of fundamental interest. The yet to be fully elucidated complexity of muscle blood flow regulation involves a balancing act between optimizing blood flow to working muscles while maintaining BP within its systemic operating limits. The quality of muscle vascular conductance, expressed quantitively as ml/min/mmHg, is a measure of this balancing act, i.e., of vascular function and the ability to meet the metabolic demands of working muscle. An easily obtainable vascular functional biomarker that uncovers impairment of muscle vascular conductance at rest could therefore serve as an attractive therapeutic and preventive target.

Our findings suggest, however, that subclinical vascular impairment can be detected more sensitively at rest. In the future, valid non-invasive measurement techniques to determine vascular function not only at rest, but under exercise conditions, will enhance our understanding of the clinical impact of an eBPR despite normal BP at rest. Until then, the SBP/MET slope might be a suitable tool to interpret the BPR in athletes and may provide a basis for future research on the prognostic impact of BPR.

Another issue to address in the future is whether the diagnostic threshold to define an eBPR might be different between athletes and the normal population. As an SBP/MET slope > 6.2 mmHg/MET was associated with a 27% higher risk of mortality over 20 years in the general population (Hedman et al. 2019), this threshold has also been adopted for athletes (Bauer et al. 2020; Keller et al. 2022). In the study of Keller et al. (Keller et al. 2022), an SBP/MET slope > 6.2 mmHg/MET was found in 39.6% of the investigated athletes, which is even higher than in our current cohort (29%). Therefore, given this relatively high prevalence, it is important to determine whether different reference values of SBP/MET slope might apply for elite athletes than for the normal population. This should be evaluated in further prospective studies. In addition, diagnostic and therapeutic approaches for isolated eBPR without arterial hypertension at rest should be developed for both athletes and the general population.

### Limitations and strengths

Our study has several limitations. The number of participants limited its statistical power to reveal other associations or to determine diagnostic thresholds. The focus on male elite mixed-sports athletes might limit extrapolation of the results to other sport disciplines, to an older age group, or to women. Further, we did not control for diet and body composition. However, we included male elite athletes of a narrow age span who did not use medication and who were free of cardiovascular diseases, and we controlled for confounders like prior prolonged exercise sessions. Furthermore, the homogeneous study cohort and the rigid design of measuring cardiovascular function must be mentioned, which strengthens our analysis.

## Conclusion

Systolic CBP measured non-invasively at rest was able to predict an eBPR, defined as an SBP/MET slope > 6.2 mmHg/MET, in male elite athletes of mixed sports. An eBPR was found to be associated with a lower performance level, highlighting the influence of vascular function on both the BPR and performance of male elite athletes.

## Data Availability

The manuscript data will not be deposited.

## Abbreviations

ACSM: American Council of Sports Medicine
Aix@75: Augmentation index corrected to 75 beats per minute
baPWV: Brachial–ankle pulse wave velocity
BMI: Body mass index
BP: Blood pressure
BPR: Blood pressure response
BSA: Body surface area
CBP: Central blood pressure
CI: Confidence interval
DBP: Diastolic blood pressure
eBPR: Exaggerated blood pressure response
ECG: Electrocardiogram
ESC: European Society of Cardiology
LA: Left atrium
LAVI: Left atrial volume index
LV: Left ventricle
LVEF: Left ventricular ejection fraction
LVMI: Left ventricular mass index
MET: Metabolic equivalent of task
OR: Odds ratio
PWV: Pulse wave velocity
RV: Right ventricle
SBP: Systolic blood pressure
SD: Standard deviation
SPAP: Systolic pulmonary artery pressure
TAPSE: Tricuspid annular plane systolic excursion
TVR: Tricuspid valve regurgitation velocity
